# Efficacy and safety of once-weekly GLP-1 receptor agonist albiglutide (HARMONY 2): 52 week primary endpoint results from a randomised, placebo-controlled trial in patients with type 2 diabetes mellitus inadequately controlled with diet and exercise

**DOI:** 10.1007/s00125-015-3795-1

**Published:** 2015-11-17

**Authors:** Michael A. Nauck, Murray W. Stewart, Christopher Perkins, Angela Jones-Leone, Fred Yang, Caroline Perry, Rickey R. Reinhardt, Marc Rendell

**Affiliations:** St Josef Hospital (Ruhr-Universität Bochum), Gudrunstr. 56, D-44791 Bochum, Germany; GlaxoSmithKline, King of Prussia, PA USA; PPD Inc., Morrisville, NC USA; Creighton University, Omaha, NE USA

**Keywords:** Albiglutide, GLP-1 agonist, Randomised controlled trial, Type 2 diabetes

## Abstract

**Aims/hypothesis:**

Additional safe and effective therapies for type 2 diabetes are needed, especially ones that do not cause weight gain and have a low risk of hypoglycaemia. The present study evaluated albiglutide as monotherapy.

**Methods:**

In this placebo-controlled study, 309 patients (aged ≥18 years) with type 2 diabetes inadequately controlled by diet and exercise and who were not using a glucose-lowering agent (HbA_1c_ 7.0–10.0% [53.00–85.79 mmol/mol], body mass index 20–45 kg/m^2^, and fasting C-peptide ≥0.26 nmol/l) were randomised (1:1:1 on a fixed randomisation schedule using an interactive voice response system) to receive once-weekly albiglutide 30 mg (*n* = 102) or 50 mg (*n* = 102) or matching placebo (*n* = 105). The study treatments were blinded to both patients and study personnel. All study data were collected at individual patient clinic visits. The primary efficacy endpoint was change in HbA_1c_ from baseline to week 52. The primary analysis was applied to the intent-to-treat population. Additional efficacy and safety endpoints were assessed.

**Results:**

At week 52, both albiglutide 30 mg and 50 mg were superior to placebo in reducing HbA_1c_. The least-squares means treatment difference from placebo was −0.84% (95% CI −1.11%, −0.58%; *p* < 0.0001) with albiglutide 30 mg and −1.04% (−1.31%, −0.77%; *p* < 0.0001) with albiglutide 50 mg. Injection-site reactions were reported more frequently with albiglutide (30 mg: 17.8%; 50 mg: 22.2%) than with placebo (9.9%). Other commonly reported adverse events included nausea, diarrhoea, vomiting and hypoglycaemia; the incidences of these were generally similar across treatment groups.

**Conclusions/interpretation:**

Albiglutide is safe and effective as monotherapy and significantly lowered HbA_1c_ levels over 52 weeks, did not cause weight gain, and had good gastrointestinal tolerability and a low rate of hypoglycaemia compared with placebo.

*Trial registration* ClinicalTrials.gov NCT00849017

*Funding* This study was sponsored by GlaxoSmithKline.

**Electronic supplementary material:**

The online version of this article (doi:10.1007/s00125-015-3795-1) contains peer-reviewed but unedited supplementary material, which is available to authorised users.

## Introduction

The medical management of patients with type 2 diabetes typically consists of diet and exercise, together with glucose-lowering medications having different mechanisms of action and possible side effects, including hypoglycaemia, weight gain, gastrointestinal (GI) disturbances, genitourinary infections and fluid retention leading to oedema and/or heart failure, which may limit their use in some individuals [1, 2]. Approximately 50% of Americans with diabetes continue to have HbA_1c_ values above 7% (53.00 mmol/mol) [3] and, while a systematic review showed there to be variability with regard to attainment of an HbA_1c_ goal of <7% (53.00 mmol/mol) among the different classes of glucose-lowering agents, glucagon-like peptide-1 (GLP-1) analogues achieved the highest percentage [4]. There remains an ongoing need for new treatments for type 2 diabetes that improve treatment efficacy and reduce patient treatment burden.

The incretin hormone GLP-1 is secreted by intestinal L cells following food ingestion. This hormone stimulates insulin release in a glucose-dependent fashion, resulting in a low risk of hypoglycaemia. In addition, native GLP-1 inhibits prostprandial glucagon release, which in turn may lead to a reduction in glucose release from the liver. Importantly, GLP-1 has also been shown to delay gastric emptying and reduce food intake, subsequently resulting in weight loss [5]. The long-acting GLP-1 receptor (GLP-1R) agonist albiglutide is made up of a GLP-1 dimer that is resistant to degradation by dipeptidyl peptidase-4 (DPP-4) fused to recombinant human albumin. Its extended duration of action (half-life of approximately 5 days) allows for once-weekly dosing [6–8]. Other studies within the HARMONY phase 3 programme have shown once-weekly albiglutide to be effective, generally safe and well tolerated in combination with other oral glucose-lowering medications [9–15].

In this 3 year phase 3 trial, we report the efficacy and safety results at the 52 week primary endpoint of once-weekly albiglutide (30 mg and 50 mg) vs placebo in patients with type 2 diabetes inadequately controlled on a regimen of diet and exercise.

## Methods

### Study design and participants

HARMONY 2 was a 3 year, randomised, placebo-controlled study evaluating the efficacy and safety of subcutaneously injected albiglutide administered as either 30 mg or 50 mg once a week. There were four study periods: screening (2 weeks); run-in/stabilisation (4 weeks); treatment (156 weeks, comprising 52 weeks for primary endpoint) and post-treatment follow-up (8 weeks).

Patients were recruited from 143 sites in the USA (133 sites; *n* = 271) and Mexico (10 sites; *n* = 38). Enrolled patients were aged ≥18 years, with type 2 diabetes uncontrolled by diet and exercise (HbA_1c_ ≥ 7.0% [≥53.00 mmol/mol] and ≤10.0% [≤85.79 mmol/mol]) and a BMI of 20–45 kg/m^2^. Key exclusion criteria included history of type 1 diabetes and recent cardiovascular and/or cerebrovascular disease (see full inclusion and exclusion criteria and a list of investigators by country in the electronic supplementary material (ESM) Methods and ESM Participating study investigators).

This study was conducted according to applicable regulatory and patient privacy requirements, good clinical practice and the Declaration of Helsinki principles. The study protocol, amendments and informed consent requiring pre-approval were reviewed and approved by a national, regional or independent ethics committee or institutional review board, in accordance with the International Conference on Harmonisation of Technical Requirements for Registration of Pharmaceuticals for Human Use Good Clinical Practice and applicable country-specific requirements. All patients provided written informed consent.

### Randomisation and masking

Random assignment of 105 patients to each of the three treatment groups in a 1:1:1 ratio by an interactive voice response system was planned. Eligible patients were stratified by screening HbA_1c_ value (<8.0% [<63.93 mmol/mol] vs ≥8.0% [≥63.93 mmol/mol]), history of myocardial infarction (MI) (yes vs no) and age (<65 years vs ≥65 years). Albiglutide and matching placebo were supplied in as a fixed-dose (30 mg or 50 mg) pen injector system designed to administer drugs via abdominal subcutaneous injection. Both albiglutide and matching placebo contained the same excipients added to either lyophilised albiglutide or matching placebo. The study treatments were blinded to both patients and study personnel.

### Procedures

Eligible patients were randomly assigned to receive either albiglutide 30 mg once weekly, albiglutide 30 mg once weekly with uptitration to 50 mg at week 12, or placebo. Standard dietary, exercise and home glucose monitoring advice for diabetic patients was provided prior to beginning randomised treatment and this was reinforced at each study centre visit through to the end-of-treatment visit. After week 2, patients who experienced persistent hyperglycaemia qualified for rescue (metformin and insulin preferred; ESM Table 1).

Major cardiovascular events (MI, stroke [including transient ischaemic attack and ischaemic neurological deficit]) and all deaths that occurred during treatment were adjudicated by a Clinical Endpoint Committee and are part of an ongoing meta-analysis. An independent, masked Pancreatitis Adjudication Committee adjudicated adverse events of suspected pancreatitis. Safety was also monitored by an independent data monitoring committee. The presence of albiglutide antibodies was assessed with a validated ELISA and their albiglutide-neutralising capacity was determined using a cell-based assay (see ESM Methods). Evaluation for systemic allergic reactions (SARs) included investigator reporting and standard Medical Dictionary for Regulatory Activities (MedDRA) queries for anaphylaxis, angioedema and severe cutaneous reaction (see ESM Methods).

### Outcomes

The primary efficacy endpoint was change in HbA_1c_ from baseline at week 52 for albiglutide vs placebo. Secondary efficacy endpoints were change in fasting plasma glucose (FPG) from baseline over time, time to hyperglycaemia rescue, the proportion of patients meeting HbA_1c_ treatment goals (<6.5% [<47.53 mmol/mol] and <7.0% [<53.00 mmol/mol]) and body weight change from baseline over time. Safety assessments included the occurrence of adverse events, serious adverse events and death. Adverse events of special interest included GI events, injection-site reactions (ISRs), cardiovascular events, hypoglycaemia [16], pancreatitis, thyroid tumours, potential SARs and immunogenicity. While the analysis of overall hypoglycaemic events was pre-specified, analysis of events that occurred pre-rescue was considered post hoc at the primary endpoint.

### Statistical analysis

Using a two-sided, two-sample *t* test and a sequential test-wise significance level of 0.05, with a minimum of 89 patients in each albiglutide group, the albiglutide vs placebo comparison had at least 91% power to reject the null hypothesis of no treatment benefit if albiglutide treatment superiority was ≥0.5% and the SD for HbA_1c_ change from baseline was ≤1.0%. To allow for a premature patient loss of up to 15%, at least 105 patients were randomly assigned to each treatment group.

The primary analysis of HbA_1c_ change from baseline at week 52 evaluated the intent-to-treat (ITT) population using an ANCOVA model with treatment group, region, history of MI and age (above/below 65 years) as factors and baseline HbA_1c_ as a continuous covariate. The ITT population included all patients who received at least one dose of the study drug, had a baseline HbA_1c_ and had at least one post-baseline HbA_1c_ assessment. Imputation for missing observations was applied to efficacy endpoints evaluated at or before week 52 using the last observation carried forward (LOCF) method. Patients rescued from hyperglycaemia or discontinued from active treatment before week 52 had their last HbA_1c_ before the occurrence carried forward for the primary analysis. A sensitivity analysis was performed using a multilevel regression model of repeated measures on change from baseline HbA_1c_ through week 52 [17].

HbA_1c_ treatment effect was evaluated as the contrasts between the groups’ least-squares means relative to placebo. The contrasts were evaluated inferentially using a two-sided *t* test and a significance level of 0.05 in a sequentially ordered analysis (albiglutide 50 mg vs placebo [superiority] then albiglutide 30 mg vs placebo [superiority]) until the first test in the order failed to reject the hypothesis.

The continuous secondary efficacy endpoints of change from baseline over time in FPG and weight were analysed analogous to the primary efficacy endpoint. The between-group differences in time to hyperglycaemia rescue were compared using pair-wise logrank tests within a Kaplan–Meier model. The treatment comparison for the proportion of patients who achieved each of the clinically meaningful HbA_1c_ response levels was analysed using non-parametric, covariance-adjusted, extended Mantel–Haenszel tests. As supportive analysis, logistic-regression models with effects for treatment and other main effect variables (region, age category, history of prior MI and baseline HbA_1c_ category) were used to quantify the observed treatment effects.

Safety analyses were applied to the safety population, which included all randomly assigned patients who received at least one dose of study treatment. Safety analyses included comparative summaries of on-therapy adverse events and rates up to 52 weeks (defined as events that occurred on-therapy or within 56 days of last dose, regardless of rescue), vital sign measurements, laboratory and physical examinations and electrocardiogram assessments.

Statistical analyses were carried out with SAS version 9.1 (SAS Institute, Cary, NC, USA).

### Role of funding source

The study sponsor participated in study design, data collection, review, analysis and report writing. All authors had full access to study data. The corresponding author reviewed the trial report (signatory investigator), had full access to study data and had final responsibility for publication submission.

## Results

**Fig. 1:**
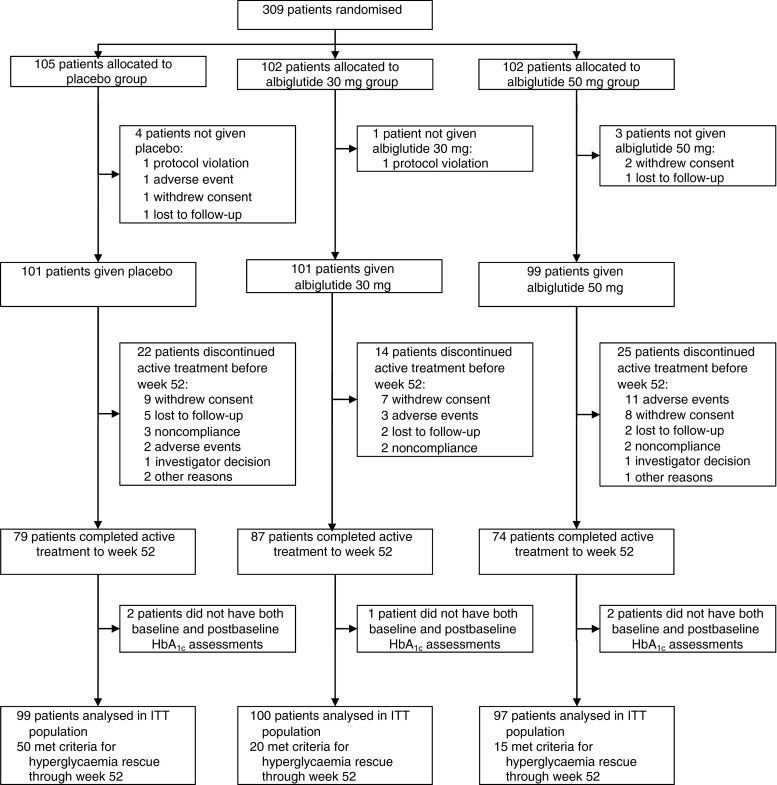
Patient disposition throughout the study. The study was ongoing at the time of data analysis and the number of patients who withdrew because of an adverse event by week 52 was reconciled to include two additional patients in each of the albiglutide treatment groups

Of the 479 patients assessed for eligibility in this study, 309 were randomly assigned to receive the following treatments: albiglutide 30 mg (*n* = 102); albiglutide 50 mg (*n* = 102) or placebo (*n* = 105) (Fig. 1). The percentage of patients continuing in the study through to week 52 included 85.3% on albiglutide 30 mg, 72.5% on albiglutide 50 mg and 75.2% on placebo (Fig. 1). Baseline demographics were similar between treatment groups (Table 1).

**Table 1:** Baseline characteristics of study participants (safety population)

Characteristic	Placebo (*n* = 101)	Albiglutide 30 mg weekly (*n* = 101)	Albiglutide 50 mg weekly (*n* = 99)
Age (years)	53.1 ± 11.7	53.6 ± 10.9	52.0 ± 11.8
Sex, male	58 (57.4)	58 (57.4)	50 (50.5)
Weight (kg)	95.4 ± 19.9	95.8 ± 19.6	97.10 ± 17.8
BMI (kg/m^2^)	33.00 ± 5.4	33.7 ± 5.1	33.9 ± 5.5
Baseline HbA_1c_ (%)	8.0 ± 0.9	8.0 ± 0.8	8.2 ± 0.9
Baseline HbA_1c_ (mmol/mol)	64.2 ± 9.9	64.5 ± 9.5	66.2 ± 10.3
Duration of diabetes (years)	4.3 ± 4.0	3.4 ± 3.7	4.2 ± 4.6
Prior MI	4 (4.0)	3 (3.0)	2 (2.0)
Race			
White	79 (78.2)	85 (84.2)	78 (78.8)
African-American/African	14 (13.9)	10 (9.9)	14 (14.1)
Asian	5 (5.0)	1 (1.0)	1 (1.0)
Ethnicity			
Hispanic/Latino	29 (28.7)	30 (29.7)	26 (26.3)

Data are mean ± SD or *n* (%)

**Fig. 2:**
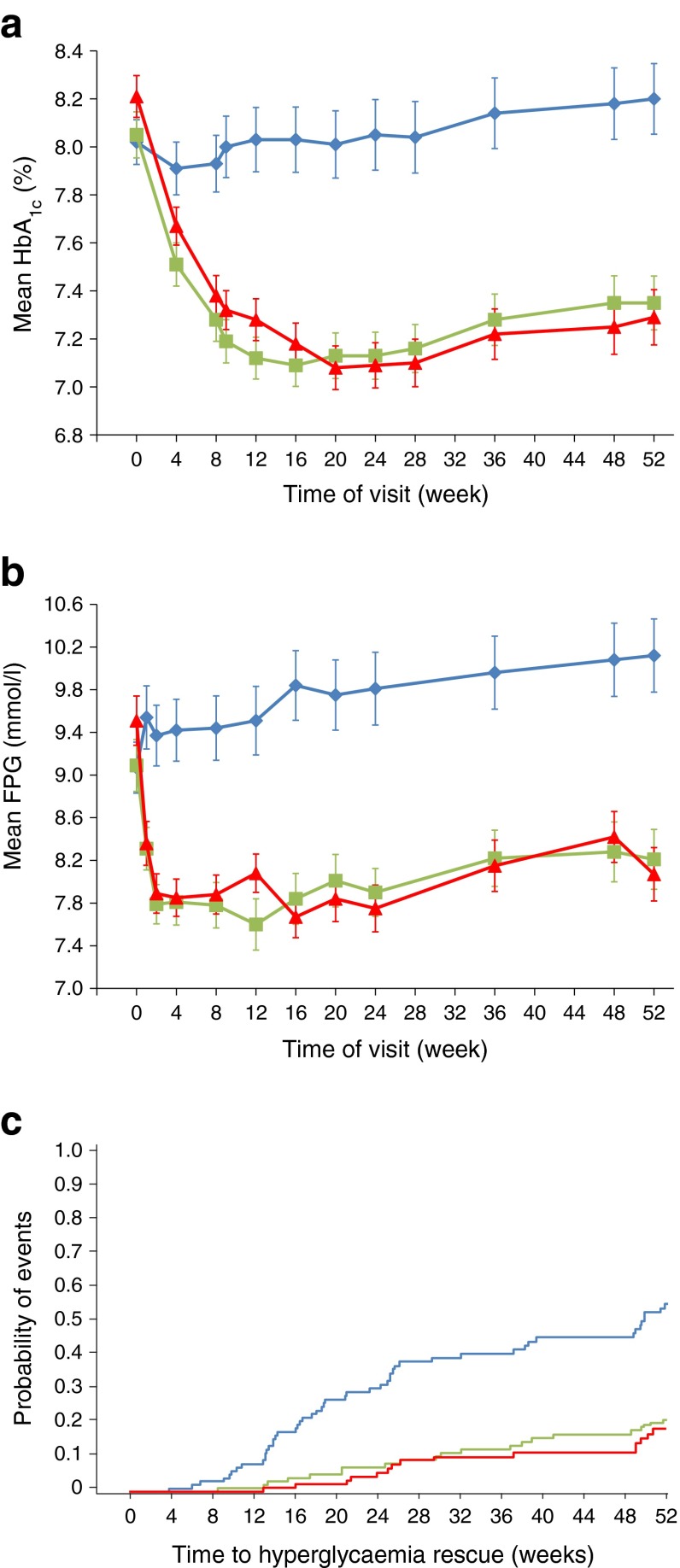
(**a**, **b**) Mean change in HbA_1c_ (**a**) and FPG (**b**) from baseline through to week 52. Data are means ± SEM. Blue diamonds, placebo (*n* = 99); green squares, albiglutide 30 mg (*n* = 100); red triangles, albiglutide 50 mg (*n* = 97) uptitration at week 12. To convert values for HbA_1c_ in DCCT % into mmol/mol, subtract 2.15 and multiply by 10.929. (**c**) Kaplan–Meier plot of probability of hyperglycaemic rescue. Blue line, placebo; green line, albiglutide 30 mg; red line, albiglutide 50 mg. HbA_1c_ and FPG analyses were for the ITT population with LOCF; Kaplan–Meier plot of probability of hyperglycaemic rescue was for the ITT population

Over 52 weeks of treatment, HbA_1c_ decreased from baseline in both albiglutide groups and increased in the placebo group (Fig. 2a). The treatment difference (albiglutide minus placebo) of the model-adjusted least-squares mean change in HbA_1c_ from baseline to week 52 (ESM Table 2) was statistically significant for both albiglutide groups (albiglutide 30 mg: −0.84% [95% CI −1.11%, −0.58%], *p* < 0.0001; albiglutide 50 mg: −1.04% [−1.31%, −0.77%], *p* < 0.0001). Post hoc sensitivity analyses of the primary efficacy endpoint were consistent with the primary efficacy results (ESM Table 2). Changes in FPG at week 52 were consistent with HbA_1c_ results (Fig. 2b). The treatment difference was statistically significant for both albiglutide groups (albiglutide 30 mg vs placebo: −1.89 mmol/l [95% CI −2.55, −1.22], *p* < 0.0001; albiglutide 50 mg vs placebo: −2.38 mmol/l [−3.05, −1.71], *p* < 0.0001). At week 52, the HbA_1c_ treatment goal of < 7.0% (53.00 mmol/mol) was met by 49.0%, 40.2% and 21.4% of patients treated with albiglutide 30 mg, albiglutide 50 mg and placebo, respectively (both *p* ≤ 0.0002) and the goal of HbA_1c_ < 6.5% (47.53 mmol/mol) was met by 25.0%, 24.7% and 10.2% of patients treated with albiglutide 30 mg, albiglutide 50 mg and placebo, respectively (both *p* < 0.005).

The difference in the time to hyperglycaemia rescue was statistically significant (Fig. 2c) in favour of each albiglutide group (albiglutide 30 mg or 50 mg) (*p* < 0.0001). At week 52, more placebo patients (50.5%) used rescue therapy than patients in either albiglutide group (albiglutide 30 mg, 20.0%; albiglutide 50 mg, 15.5%). Up to week 52, rescue probability did not exceed 21.7% with albiglutide 30 mg and 18.6% with albiglutide 50 mg, while the probability with placebo was as high as 55.7%. Among patients requiring rescue medication, metformin was the most commonly used; sulfonylureas (glibenclamide, glimepiride or glipizide), insulin glargine (A21Gly,B31Arg,B32Arg human insulin) and sitagliptin were used by a small proportion of patients.

Weight loss was not statistically significantly different when comparing the placebo and albiglutide groups at week 52 (least-squares mean change from baseline −0.39 kg with albiglutide 30 mg, −0.86 kg with albiglutide 50 mg and −0.66 kg with placebo).

For the safety profile at week 52, the proportion of patients experiencing adverse events was higher with albiglutide 30 mg and albiglutide 50 mg than with placebo (Table 2). More albiglutide-treated patients withdrew from the study because of adverse events, the main reasons in the 50 mg group being GI events (*n* = 3) and ISRs (*n* = 4). However, 5 of the 13 patients in the albiglutide 50 mg group were on the 30 mg dose at the time of withdrawal. The incidence of serious adverse events (fatal and non-fatal) at week 52 was similar across the two albiglutide treatment groups and higher than in the placebo group; there were no on-therapy serious adverse events reported by more than one patient in any treatment group. A total of three deaths was reported up to week 52 (all in the albiglutide 50 mg group)—one due to drowning and two due to cancer (B-cell lymphoma and lung adenocarcinoma)—none of which were considered by the investigator to be related to the study drug. There were no reports of acute pancreatitis or thyroid cancer during this study. Only small changes in the incidence of adverse events, including serious adverse events, were observed when comparing on-therapy adverse events overall with on-therapy adverse events prior to rescue from hyperglycaemia with other glucose-lowering medications (mostly metformin) that were added to albiglutide (ESM Table 3).

**Table 2:** On-therapy adverse events at week 52 (safety population)

Event	Placebo (*n* = 101)	Albiglutide 30 mg weekly (*n* = 101)	Albiglutide 50 mg weekly (*n* = 99)
Overall (*n*/%/rate^a^)			
Any adverse event	77/76.2/329	79/78.2/411	81/81.8/349
Serious adverse event	8/7.9/7.9	11/10.9/13.2	10/10.1/11.4
Related adverse event	21/20.8/60.3	35/34.7/120	36/36.4/94.1
Adverse event leading to withdrawal	2/2.0/2.0	5/5.0/4.7	13/13.1/13.4
Most common adverse event (≥6.0% in either albiglutide group), by preferred term (*n*/%/rate^a^)			
Injection-site reaction	2/2.0/29.6	9/8.9/35.6	14/14.1/34.0
Diarrhoea	12/11.9/14.8	10/9.9 /15.0	13/13.1/15.5
Nausea	8/7.9/7.9	10/9.9/11.3	9/9.1/10.3
Upper respiratory tract infection	10/9.9/10.9	6/5.9/5.6	9/9.1/11.4
Nasopharyngitis	6/5.9/5.9	6/5.9/7.5	7/7.1/7.2
Sinusitis	2/2.0/2.0	3/3.0/2.8	7/7.1/10.3
Urinary-tract infection	3/3.0/5.9	1/1.0/0.9	6/6.1/8.3
Headache	14/13.9/18.7	10/9.9/15.0	6/6.1/8.3
GI adverse event (*n*/%/rate^a^)			
Any event	27/26.7/41.4	32/31.7/49.7	30/30.3/51.6
Gastro-oesophageal reflux disease	2/2.0/2.0	1/1.0/0.9	4/4.0/4.1
Constipation	3/3.0/3.0	2/2.0/1.9	3/3.0/3.1
Vomiting	1/1.0/1.0	3/3.0/2.8	3/3.0/4.1
Dyspepsia	3/3.0/3.0	2/2.0/2.8	1/1.0/1.0
Pre-rescue hypoglycaemic event (*n*/%/rate^b^)			
Any hypoglycaemic event	4/4.0/5.65	6/5.9/9.46	6/6.1/10.09
Severe	0/0/0	0/0/0	0/0/0
Documented symptomatic	2/2.0/2.83	1/1.0/2.10	0/0/0
ISR, *n* (%)			
Any ISR	10 (9.9)	18 (17.8)	22 (22.2)
Mild ISR event^c^	44 (100)	82 (90)	49 (89)
Withdrawal due to an ISR	0 (0)	4 (4.0)	4 (4.0)
No. of patients with one or two ISR events among patients with an ISR	8/10 (80)	9/18 (50)	15/22 (68)

^a^Event rate per 100 patient-years

^b^Event rate per patient-year. American Diabetes Association criteria [16]: Severe—event requiring another person to administer a resuscitative action; Documented symptomatic—plasma glucose concentration ≤3.9 mmol/l (70 mg/dl) and presence of hypoglycaemic symptoms. While analysis of overall hypoglycaemic events was pre-specified, analysis of events that occurred pre-rescue was considered post hoc at the primary endpoint; the number of patients with one or two ISR events was also considered post hoc

^c^The bracketed numbers are percentages of mild ISR events with total ISR events as the denominator

**Fig. 3:**
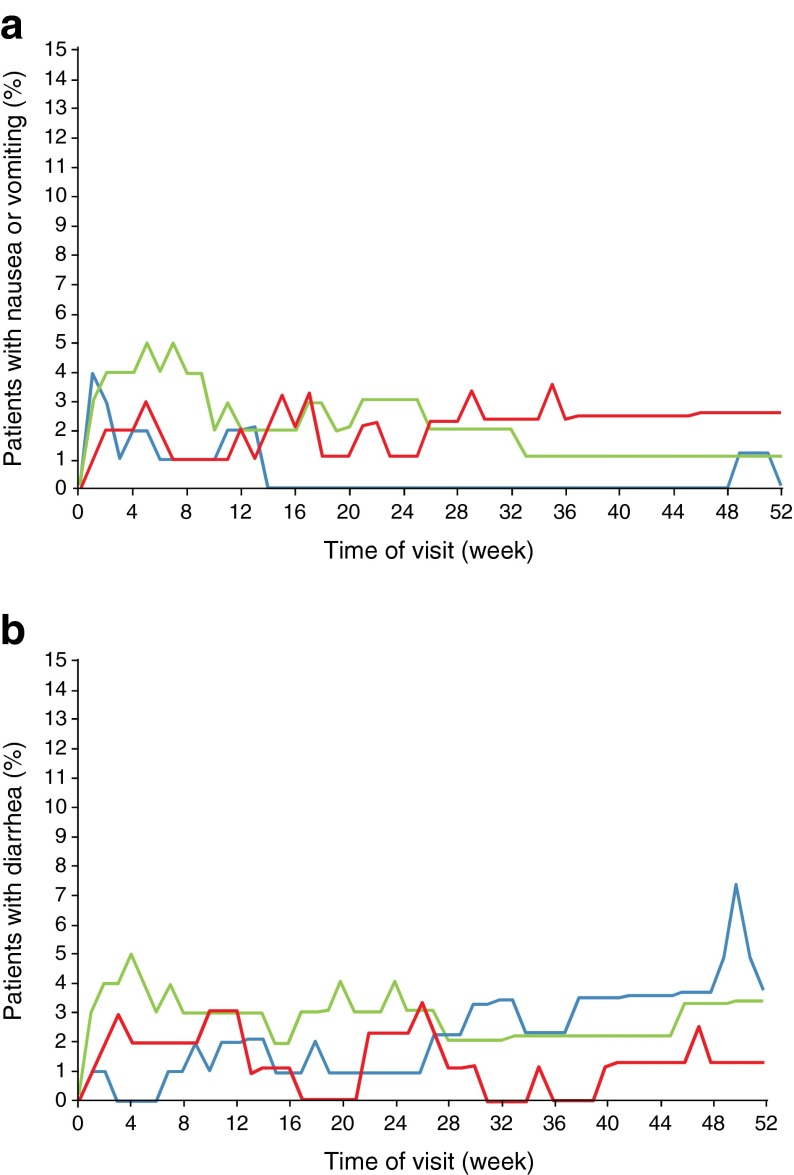
Nausea/vomiting (**a**) and diarrhoea events (**b**) over time to week 52. Analyses are for the safety population, defined as all randomised patients who received at least one dose of study drug. Blue lines, placebo (*n* = 101); green lines, albiglutide 30 mg (*n* = 101); red lines, albiglutide 50 mg (*n* = 99) uptitration at week 12

The most common adverse events reported with albiglutide were GI events and ISRs. The frequency of all GI events was similar across groups: 31.7% and 30.3% with albiglutide 30 mg and 50 mg, respectively, and 26.7% with placebo. The reported incidence of nausea, diarrhoea, and constipation events was similar among the treatment groups (Table 2), and the incidence of vomiting was low in all groups but higher with albiglutide 30 mg and 50 mg (3.0% in each) than with placebo (1.0%). The combined frequency of nausea or vomiting rose to a peak (4–5%) within the first weeks after initiation of albiglutide and thereafter fluctuated between 1% and 3% at any given time point up to week 52 (Fig. 3a). A similar pattern was observed for diarrhoea, which rose to a peak (3–5%) within the first weeks after initiation of albiglutide and thereafter fluctuated between 0% and 4% over 52 weeks (Fig. 3b). No dose–response relationship was observed for the 30 mg and 50 mg curves for any of these GI events.

ISRs (most commonly reported as a reaction, erythaema, rash or haematoma at the injection site) were reported more frequently with both albiglutide dose groups than with placebo (Table 2), but a dose–response relationship was not observed. Among patients with ISRs in the albiglutide groups, 50–69% of patients experienced one or two events, most had mild events and the withdrawal rate was low (4%), although higher than placebo (0%).

Documented symptomatic hypoglycaemia (≤3.9 mmol/l [70 mg/dl]) occurred in one patient treated with albiglutide 30 mg; there were no reports of severe hypoglycaemia (Table 2 and ESM Table 4).

The incidence and total number of investigator-assessed cardiovascular adverse events were lower in the albiglutide 50 mg group (8.1%, 12 events) than in the albiglutide 30 mg (16.8%, 28 events) and placebo groups (16.8%, 23 events). Small trends for lower blood pressure were observed, and heart rate changes from baseline in the albiglutide treatment groups compared with placebo showed increases of 1–2 beats per min (ESM Table 5).

There were no reports of serious events of angioedema or anaphylaxis during this study (see Systemic allergic reactions in the ESM Methods). The proportion of patients who developed anti-albiglutide antibodies was low (5.5% at any visit) and none of the antibodies detected were neutralising. Assessment of data for haematology, serum chemistry, lipid profile, vital signs, electrocardiogram readings and physical examinations generally produced unremarkable findings.

## Discussion

It is important to conduct studies of monotherapy in patients with type 2 diabetes because they provide evidence of a drug’s safety and efficacy without the influence of other glucose-lowering drugs. Our findings showed that albiglutide 30 mg and 50 mg administered once weekly provided a rapid, clinically relevant and durable glycaemic lowering through to the primary endpoint at week 52. Results for the secondary endpoints (FPG, HbA_1c_ targets and time to hyperglycaemic rescue) were supportive of the primary endpoint. Modest weight loss was observed in all groups, including placebo, and a low hypoglycaemic potential was demonstrated.

The placebo-subtracted reduction in HbA_1c_ observed with the albiglutide 50 mg dose in this study was comparable with results from liraglutide and dulaglutide placebo-controlled monotherapy studies and was greater than the reduction observed with exenatide and lixisenatide [18–21]. Albiglutide treatment produced a rapid effect on FPG; there was a steep decline from baseline up to week 2 and the reduced levels were maintained through to week 52. These data are further supported by albiglutide’s sustained effect, as demonstrated by the lower proportion of patients who required rescue from hyperglycaemia in the albiglutide vs placebo groups and by the proportion of patients treated with both doses of albiglutide who achieved a treatment goal of HbA_1c_ <7% (<53.00 mmol/mol). A greater proportion of patients receiving 30 mg albiglutide achieved an HbA_1c_ <7%, which may have been due to the higher starting HbA_1c_ value in patients in the group receiving the 50 mg dose.

Molecular size and other properties may have an impact on the effect relative to other compounds in the central nervous system. As a large albumin-based molecule, albiglutide (approximately 72 kDa) may not readily cross the blood–brain barrier or diffuse into the brain at the area postrema (vomiting centre) or hypothalamus (centre for the regulation of appetite and food intake) where there is a breakdown in the blood–brain barrier and thus it may have a more restricted access to the central nervous system than the smaller GLP-1 peptides. This may explain the finding that weight loss with albiglutide did not differ significantly from that with placebo and is an area where further research is needed.

With few exceptions, the safety profile was comparable across the three treatment groups over 52 weeks. Consistent with the known profile of GLP-1 agonists [22], GI events were among the most common adverse events in the albiglutide groups. However, diarrhoea and nausea were also among the most common in the placebo group. Additionally, the frequency of nausea and vomiting reported with albiglutide in this study are consistent with those reported in other albiglutide studies [6–8] and were generally lower than has been reported in studies with liraglutide and exenatide [23]. Moreover, in clinical trials of albiglutide that included exenatide [8] and liraglutide [9] comparator arms the incidence of GI adverse events was lower with albiglutide compared with the other GLP-1R agonists. These differences in GI tolerability profiles may be due in part to differences in the time taken to reach maximum blood concentration (3–4 days for albiglutide; a matter of hours for both exenatide and liraglutide [24, 25]). Furthermore, the large molecular size of albiglutide and its likely inability to cross the blood–brain barrier may also account for the difference in GI tolerability when comparing albiglutide with other members of its class. Both efficacy measurements and adverse events have been partly linked to centrally mediated effects [26, 27].

In this current study, although ISRs occurred with both placebo and albiglutide, the incidence was higher with albiglutide. However, events were generally mild and mostly did not lead to withdrawal from the study. Since incretin-based therapies have a glucose-dependent mechanism of action, albiglutide was expected to have a low hypoglycaemic potential and this was further supported by data from this study.

When considering differences between the 30 mg and 50 mg doses of albiglutide, a dose–response relationship was observed for glycaemic variables but the adverse event profiles were comparable with no dose-related trends. A partial explanation for the latter finding could be tolerance or tachyphylaxis to some reported adverse events. Although it is clinically important to assess differences between the 30 mg and 50 mg doses of albiglutide, this study was not powered to do so.

A limitation of this study was the omission of a clinically relevant comparator. However, the comparison against placebo provided the best data with which to test the effectiveness and tolerability of albiglutide alone. Second, the conclusions from this study are limited to the population studied and might not be applicable to the general population of patients with type 2 diabetes.

Our findings demonstrated that albiglutide as monotherapy for 52 weeks had a favourable benefit–risk profile in patients with type 2 diabetes, provided durable glycaemic control, did not promote weight gain and was generally well tolerated. The complete 3 year results for this study will further clarify the results reported here.

## Electronic supplementary material

ESM Methods (PDF 80 kb)

ESM Table 1 (PDF 34 kb)

ESM Table 2 (PDF 51 kb)

ESM Table 3 (PDF 49 kb)

ESM Table 4 (PDF 34 kb)

ESM Table 5 (PDF 46 kb)

ESM Participating Study Investigators by Country (PDF 28 kb)

## Abbreviations

FPG: Fasting plasma glucose
GI: Gastrointestinal
GLP-1: Glucagon-like peptide-1
ISR: Injection-site reaction
ITT: Intent-to-treat
LOCF: Last observation carried forward
MI: Myocardial infarction
SAR: Systemic allergic reaction
