# Supplementary material for: Efficacy and safety of once-weekly GLP-1 receptor agonist albiglutide (HARMONY 2): 52 week primary endpoint results from a randomised, placebo-controlled trial in patients with type 2 diabetes mellitus inadequately controlled with diet and exercise
Source: Diabetologia. 2015 Nov 17;59:266–74. doi: 10.1007/s00125-015-3795-1 (PMC4705137; doi:10.1007/s00125-015-3795-1)
Supplement: Supplementary file 1 — (PDF 80 kb) [file 125_2015_3795_MOESM1_ESM.pdf]

ESM METHODS

Antibody Detection Assays

The detection assays are ELISA based, which include screening, confirmation, and titration assays. The procedure for the albiglutide immunoglobulin (Ig) (GAM) ELISA is described below:

1. 96-well ELISA plates are coated with albiglutide overnight.
2. Samples are diluted 1:15 or 1:30 and incubated on the blocked plates for 3 hours.
3. A horseradish peroxidase-labeled antihuman Ig (GAM) antibody is used as a detection antibody.
4. A tetramethylbenzidine substrate is used to produce the enzymatic reaction, which is followed by an acid stop procedure.
5. The plates are read at 450 nm using a SpectraMax Plus 384 reader (Molecular Devices, Inc, Sunnyvale, California).
6. A positive control (positive control, normal serum pool spiked with anti-GLP-1 antibody) and negative control (negative control, pool normal human sera) are included on each assay plate.
7. Relative optical density values are calculated by dividing the mean optical density of sample or positive control by the mean optical density of the negative control.

Neutralizing Antibody Assays

The neutralizing antibody assays are cell-based assays, which include screening, confirmation, and titration assays. Albiglutide-neutralizing activity is measured by adding serum samples preincubated with a fixed concentration of albiglutide to GLP-1 receptor/CRE (cyclic adenosine

monophosphate response element)-luciferase transfected cells and measuring luminescence intensity after 3 hours of incubation. Samples possessing neutralizing activity will generate a reduced signal and will be further tested in the confirmation (specificity) assay if the generated signal is positive (ie, below the assay's screening cutoff).

Inclusion/Exclusion Criteria

Subjects eligible for enrollment in the study must meet all of the following criteria:

1. Male or female, 18 years of age or older, with a historical diagnosis of type 2 diabetes mellitus who are experiencing inadequate glycemic control and receiving no current antidiabetic therapy. The subject should not have received >7 contiguous days of any antidiabetic agent within the 3 months before Screening
2. Failed treatment with diet and exercise for at least 1 month before randomization
3. Diagnosis of type 2 diabetes must be based on current American Diabetes Association criteria
4. BMI $\geq$20 kg/m^2 and $\leq$45 kg/m^2
5. Fasting C-peptide $\geq$0.8 ng/mL ($\geq$0.26 nmol/L)
6. HbA_{1c} between 7.0% and 10.0% [53.00 mmol/mol and 85.79 mmol/mol], inclusive, at Visit 5 (Week -1). The HbA_{1c} value may be checked up to 4 times, and if the average of these determinations meets the criterion, the subject may be randomly assigned to treatment
7. For the regular use of other medications (does not include medications excluded by the protocol [see Section 5.6.2, for example, weight loss medications are excluded]), it is preferred that the subjects are receiving a stable dose for at least 4 weeks before Screening; however, as necessary during the Run-in/Stabilization Period and the Treatment Period,

prescription or over-the-counter medications are allowed and may be adjusted by the investigator to optimize treatment (e.g., increase or decrease of medication to treat blood pressure or hyperlipidemia in accordance with accepted local medical practice and relevant guidance documents)

8. Use of oral or systemically injected glucocorticoids is generally not allowed within the 3 months before randomization; however, short courses of oral steroids (single dose or multiple doses for up to 2 days) may be permitted provided these cases are discussed with the medical monitor. Inhaled, intra-articular, and topical corticosteroids are allowed
9. Hemoglobin ≥11 g/dL (≥110 g/L) for male subjects and ≥10 g/dL (≥100 g/L) for female subjects
10. Creatinine clearance >60 mL/min (calculated using the Cockcroft-Gault formula)
11. Thyroid-stimulating hormone level is normal or clinically euthyroid as demonstrated by further thyroid tests (e.g., T4, T3, thyroid-binding globulin)
12. Female subjects of childbearing potential (i.e., not surgically sterile and/or not postmenopausal) must be practicing adequate contraception. Methods of adequate contraception include the following: abstinence, injectable progestogen, implants of levonorgestrel, estrogenic vaginal ring, percutaneous contraceptive patches, intrauterine device or intrauterine system, male partner sterilization (vasectomy with documentation of azoospermia) before the female subjects entry into the study and this male partner is the sole partner for that subject, double-barrier method (condom and occlusive cap plus nonoxynol-9), or oral contraceptives in combination with a second method of contraception (e.g., condom and occlusive cap). Adequate contraception must be practiced for the duration of participation in the study including the 8-week Posttreatment Follow-up Period

13. Able and willing to monitor his/her own blood glucose concentrations with a home glucose monitor
14. No major illness or debility that in the investigator's opinion prohibits the subject from completing the study
15. Able and willing to provide written informed consent

Subjects eligible for randomization in the study must meet the following additional criterion:

- HbA_{1c} concentration between 7.0% and 10.0% [53.00 mmol/mol and 85.79 mmol/mol], inclusive, after the Run-in/Stabilization Period. If the subject does not qualify for randomization based on this criterion, the assessment may be repeated on a weekly basis for a maximum of 4 additional weeks before randomization. The mean of all HbA_{1c} assessments must be between 7.0% and 10.0% (53.00 mmol/mol and 85.79 mmol/mol), inclusive, for the subject to be eligible for randomization
- If the mean of the 3 screening ECGs for the QTc interval (Fridericia) is >470 ms, 3 repeat ECGs may be obtained during the Run-in/Stabilization Period. The repeat ECGs will be overread by the central reader (eResearch Technologies, Inc). If the mean QTc interval (Fridericia) for all 6 ECGs is ≤470 ms, the subject qualifies for entry into the study (the ECG equipment provided to the investigator reports the QTc as both Bazett and Fridericia corrections). At Baseline, the subject's ECGs should be reviewed by the investigator before the administration of the investigational product.
- If the lipase result is above the ULN, then the subject will not be randomly assigned to treatment. If the amylase result is above the ULN and the lipase is below the ULN, then an isoenzyme analysis will be done by the central laboratory. If this additional test confirms

that the pancreatic isoenzyme fraction is not the cause of the elevation, the subject may continue in the study.

Subjects meeting any of the following criteria must not be enrolled in the study:

1. History of cancer, other than squamous cell or basal cell carcinoma of the skin, that has not been in full remission for at least 3 years before Screening. (A history of treated cervical intraepithelial neoplasia I or cervical intraepithelial neoplasia II is allowed)
2. History of treated diabetic gastroparesis
3. Current ongoing symptomatic biliary disease or history of pancreatitis
4. History of significant gastrointestinal surgery, including gastric bypass and banding, antrectomy, Roux-en-Y bypass, gastric vagotomy, small bowel resection, or surgeries thought to significantly affect upper gastrointestinal function
5. Recent (as defined below) clinically significant cardiovascular and/or cerebrovascular disease including, but not limited to, the following:
    - Previous history of stroke or transient ischemic attack within 1 month before Screening. However, subjects who are deemed clinically stable by the investigator may be enrolled 1 month after the cerebrovascular event.
    - Acute coronary syndrome, which includes the following:
    - Documented MI within the 2 months before Screening and during the period up until receiving the first dose of study medication
    - Any cardiac surgery including percutaneous transluminal coronary angioplasty, coronary stent placement, or coronary artery bypass graft surgery within the 2 months

before Screening and during the period up until receiving the first dose of study medication

- Unstable angina not responsive to nitroglycerin within the 2 months before Screening and during the period up until receiving the first dose of study medication
- Unstable cardiac rhythm; controlled atrial fibrillation is allowed
- Current or history of heart failure (New York Heart Association class III or IV)
- Resting systolic pressure is >160 mm Hg and/or diastolic pressure >100 mm Hg. If the subject's systolic blood pressure >160 mm Hg or the subject's diastolic blood pressure is >100 mm Hg at Screening, the blood pressure readings may be repeated at 5-minute intervals for a total of 3 determinations. If the average of the systolic or diastolic pressure readings still does not meet the criteria, the subject can be treated and rescreened. It is preferred that subjects be on a stable dose of medication for at least 4 weeks before being rescreened; however, when stable, they may be rescreened at the discretion of the investigator

  Should a subject not meet this criterion on Visit 6 (first dose of study medication following the randomization visit), the subject may continue in the study at the discretion of the investigator with the understanding that the subject's hypertension will be monitored and treated in accordance with accepted local medical practice and relevant guidance documents
- Mean QTc interval (Fridericia) >470 ms confirmed by a central reader at Screening

6. Hemoglobinopathy that may affect determination of HbA_{1c}
7. History of human immunodeficiency virus infection

8. History of total bilirubin >1.5 × ULN unless the subject has a previously known history of Gilbert's syndrome and a fractionated bilirubin that shows conjugated bilirubin <35% of total bilirubin
9. ALT or aspartate aminotransferase (AST) >2.5 × ULN
10. Fasting triglyceride level >850 mg/dL at Screening or Week -1 (Visit 5). If the subject's triglyceride level is >500 mg/dL at Screening and Week -1, the subject is excluded. If the subject meets the aforementioned exclusion criteria for triglycerides, the subject can be treated and rescreened. Treated subjects must be on a stable dose of medication for at least 4 weeks before being rescreened
11. Acute symptomatic (within 3 months before Screening) infection with hepatitis B; however, subjects with past or chronic hepatitis B or hepatitis C are allowed provided the requirements for ALT, AST, and total bilirubin are met
12. History of a psychiatric disorder that will affect the subject's ability to participate in the study
13. History of alcohol or substance abuse within 1 year before Screening
14. Positive urine drug screen result at Screening, unless the subject is taking a medically approved medication for which a positive drug screen simply verifies the use of this medication
15. Female subject is pregnant (confirmed by laboratory testing), lactating, or <6 weeks postpartum
16. Known allergy to any GLP-1 analogue, insulin, other study medication's excipients, excipients of albiglutide, or Baker's yeast

17. Receipt of any investigational drug within the 30 days or 5 half-lives, whichever is longer, before Screening, a history of receipt of an investigational antidiabetic drug within the 3 months before randomization, or receipt of albiglutide in previous studies
18. History of type 1 diabetes, diabetic complications (e.g., active proliferative retinopathy or severe diabetic neuropathy) that in the opinion of the investigator would preclude effective participation in the study, or a history of ketoacidosis or hyperosmolar coma
19. Contraindications (as per the prescribing information) for the use of either background or potential randomized study medications
20. History of or family history of medullary carcinoma
21. History of or family history of multiple endocrine neoplasia type 2

Systemic Allergic Reactions

An evaluation for systemic allergic reactions (SARs) using the standard MedDRA queries for angioedema, anaphylaxis, and severe cutaneous reaction found two events (urticaria in one albiglutide 50-mg patient and laryngeal edema in one placebo patient); both events were nonserious, mild in intensity, and neither led to withdrawal. None of these patients had a positive antibody result. No events in either treatment group were reported as angioedema or anaphylaxis.
