# Supplementary material for: Efficacy and safety of once-weekly GLP-1 receptor agonist albiglutide (HARMONY 2): 52 week primary endpoint results from a randomised, placebo-controlled trial in patients with type 2 diabetes mellitus inadequately controlled with diet and exercise
Source: Diabetologia. 2015 Nov 17;59:266–74. doi: 10.1007/s00125-015-3795-1 (PMC4705137; doi:10.1007/s00125-015-3795-1)
Supplement: Supplementary file 2 — (PDF 34 kb) [file 125_2015_3795_MOESM2_ESM.pdf]

ESM Table 1. Hyperglycemia rescue criteria

Time interval on treatment	Hyperglycemia rescue
≥Day 1 and <week 2	No rescue
≥Week 2 and <week 4	A single FPG ≥15.5 mmol/l (≥280 mg/dl)[a]
≥Week 4 and <week 12	A single FPG ≥13.9 mmol/l (≥250 mg/dl)[a]
≥Week 12 and <week 24	HbA_{1c} ≥8.5% [69.39 mmol/mol] and ≤0.5% reduction from baseline
≥Week 24 and <week 48	HbA_{1c} ≥8.5% [69.39 mmol/mol]
≥Week 48 and <week 156	HbA_{1c} ≥8.0% [63.93 mmol/mol]

[a]Confirmed by a second sample collected within 7 days analyzed by the central laboratory.

FPG, fasting plasma glucose, HbA_{1c}, glycosylated hemoglobin
