# Supplementary material for: Efficacy and safety of once-weekly GLP-1 receptor agonist albiglutide (HARMONY 2): 52 week primary endpoint results from a randomised, placebo-controlled trial in patients with type 2 diabetes mellitus inadequately controlled with diet and exercise
Source: Diabetologia. 2015 Nov 17;59:266–74. doi: 10.1007/s00125-015-3795-1 (PMC4705137; doi:10.1007/s00125-015-3795-1)
Supplement: Supplementary file 3 — (PDF 51 kb) [file 125_2015_3795_MOESM3_ESM.pdf]

ESM Table 2. Change in HbA_{1c} from baseline at week 52 (ITT population)

	Placebo (*n* = 99)	Albiglutide 30 mg weekly (*n* = 100)	Albiglutide 50 mg weekly (*n* = 97)
**HbA_{1c} (%) [mmol/mol]**			
Baseline, mean (SD)	8.02 (0.908) [64.15 (9.93)]	8.05 (0.867) [64.47 (9.48)]	8.21 (0.942) [66.22 (10.30)]
Week 52, mean (SD)	8.20 (1.458) [66.11 (15.94)]	7.35 (1.150) [56.82 (12.57)]	7.29 (1.104) [56.17 (12.07)]
**Model-adjusted change from baseline[a]**			
% difference (SE)	0.15% (0.097)	−0.70% (0.096)	−0.89% (0.097)
**Difference from placebo**			
% difference of least-squares means		−0.84%	−1.04%
95% CI		(−1.11 to 0.58)	(−1.31 to −0.77)
Superiority *P* value		<0.0001	<0.0001
**Sensitivity analysis[b]**			
**Model-adjusted change from baseline[c]**			
% difference (SE)	−0.15% (0.091)	−0.85% (0.077)	−1.09% (0.081)
**Difference from placebo**			
% difference of least-squares means		−0.70%	−0.94%
95% CI		(−0.94 to −0.47)	(−1.18 to 0.70)

Superiority *P* value		<0.0001	<0.0001

HbA_{1c}, glycosylated hemoglobin; SD, standard deviation; SE, standard error. [a]Adjusted for treatment group, region, history of prior myocardial infarction, and age category as factors and baseline HbA_{1c}. [b]Sensitivity analyses were performed post hoc at the primary endpoint. [c]Based on mixed-model repeated measured analysis.
