# Supplementary material for: Efficacy and safety of once-weekly GLP-1 receptor agonist albiglutide (HARMONY 2): 52 week primary endpoint results from a randomised, placebo-controlled trial in patients with type 2 diabetes mellitus inadequately controlled with diet and exercise
Source: Diabetologia. 2015 Nov 17;59:266–74. doi: 10.1007/s00125-015-3795-1 (PMC4705137; doi:10.1007/s00125-015-3795-1)
Supplement: Supplementary file 4 — (PDF 49 kb) [file 125_2015_3795_MOESM4_ESM.pdf]

ESM Table 3. On-therapy adverse events to week 52 and prior to hyperglycemic rescue (safety population)

	Placebo<br>(*n* = 101)	Albiglutide<br>30 mg weekly<br>(*n* = 101)	Albiglutide<br>50 mg weekly<br>(*n* = 99)
	n / % / rate*	n / % / rate*	n / % / rate*
**Most common adverse events (≥6.0% in either albiglutide group), by preferred term**			
Injection-site reaction	2 / 2.0 / 42.4	9 / 8.9 / 39.9	14 / 14.1 / 37.0
Diarrhea	4 / 4.0 / 5.7	9 / 8.9 / 14.7	11 / 11.1 / 13.5
Nausea	6 / 5.9 / 8.5	10 / 9.9 / 12.6	9 / 9.1 / 11.2
Upper respiratory tract infection	9 / 8.9 / 12.7	5 / 5.0 / 5.3	8 / 8.1 / 10.1
Nasopharyngitis	5 / 5.0 / 7.1	6 / 5.9 / 7.4	6 / 6.1 / 6.7
Sinusitis	2 / 2.0 / 2.8	3 / 3.0 / 3.2	7 / 7.1 / 10.1
Urinary tract infection	3 / 3.0 / 8.5	0 / 0 / 0	6 / 6.1 / 9.0
Headache	13 / 12.9 / 22.6	10 / 9.9 / 16.8	5 / 5.1 / 7.9
**Gastrointestinal adverse events**			
Any event	20 / 19.8 / 35.3	29 / 28.7 / 51.5	27 / 27.3 / 50.4
Gastroesophageal reflux disease	1 / 1.0 / 1.4	1 / 1.0 / 1.1	4 / 4.0 / 4.5
Constipation	3 / 3.0 / 4.2	1 / 1.0 / 1.1	3 / 3.0 / 3.4
Vomiting	1 / 1.0 / 1.4	3 / 3.0 / 3.2	3 / 3.0 / 4.5
Dyspepsia	3 / 3.0 / 4.2	1 / 1.0 / 2.1	1 / 1.0 / 1.1

[a]Event rate per 100 patient-years.
