# Supplementary material for: Efficacy and safety of once-weekly GLP-1 receptor agonist albiglutide (HARMONY 2): 52 week primary endpoint results from a randomised, placebo-controlled trial in patients with type 2 diabetes mellitus inadequately controlled with diet and exercise
Source: Diabetologia. 2015 Nov 17;59:266–74. doi: 10.1007/s00125-015-3795-1 (PMC4705137; doi:10.1007/s00125-015-3795-1)
Supplement: Supplementary file 5 — (PDF 34 kb) [file 125_2015_3795_MOESM5_ESM.pdf]

ESM Table 4. Hypoglycemia events through week 52 and prior to hyperglycemic rescue (safety population)

	Placebo (n = 101)		Albiglutide 30 mg weekly (n = 101)		Albiglutide 50 mg weekly (n = 99)	
	n / % / rate[a]	No. of events	n / % / rate[a]	No. of events	n / % / rate[a]	No. of events
Any hypoglycemic event	4 / 4.0 / 5.65	4	6 / 5.9 / 9.46	9	6 / 6.1 / 10.09	9
Severity[b]						
Severe	0	0	0	0	0	0
Documented symptomatic	2 / 2.0 / 2.83	2	1 / 1.0 / 1.10	1	0	0
Asymptomatic	0	0	0	0	2 / 2.0 / 2.24	2
Probable symptomatic	2 / 2.0 / 2.83	2	5 / 5.0 / 5.31	7	2 / 2.0 / 2.24	2
Relative	0		1 / 1.0 / 1.05	1	4 / 4.0 / 5.61	5

[a]Event rate per 100 patient–years. [b]American Diabetes Association criteria (13). Severe—event requiring another person to administer a resuscitative action; Documented symptomatic—plasma glucose concentration ≤3.9 mmol/l (70 mg/dl) and presence of hypoglycemic symptoms; Asymptomatic—plasma glucose concentration ≤3.9 mmol/l (70 mg/dl) and no hypoglycemic symptoms; Probable symptomatic—typical hypoglycemic symptoms but plasma glucose concentration not measured; Relative—plasma glucose concentration >3.9 mmol/l (70 mg/dl) and typical hypoglycemic symptoms.

Note: While analysis of overall hypoglycemic events was prespecified, analysis of events that occurred pre-rescue was considered post hoc at the primary endpoint.
