# Supplementary material for: Efficacy and safety of once-weekly GLP-1 receptor agonist albiglutide (HARMONY 2): 52 week primary endpoint results from a randomised, placebo-controlled trial in patients with type 2 diabetes mellitus inadequately controlled with diet and exercise
Source: Diabetologia. 2015 Nov 17;59:266–74. doi: 10.1007/s00125-015-3795-1 (PMC4705137; doi:10.1007/s00125-015-3795-1)
Supplement: Supplementary file 6 — (PDF 46 kb) [file 125_2015_3795_MOESM6_ESM.pdf]

ESM Table 5. Change from baseline to week 52 in vital signs, lipid parameters, and calcitonin (safety population)

	**Placebo (*n* = 101)**	**Albiglutide 30 mg weekly (*n* = 101)**	**Albiglutide 50 mg weekly (*n* = 99)**
Systolic (mm Hg)	1.3 (13.09)	−2.8 (12.14)	−1.3 (13.37)
Diastolic (mm Hg)	0.1 (9.17)	−0.8 (8.21)	−0.8 (8.95)
Heart rate (bpm)	0.8 (8.92)	2.5 (8.44)	0.8 (9.00)
Total cholesterol (mmol/L)	−0.057 (1.0454)	−0.312 (0.8948)	−0.101 (0.6237)
LDL (mmol/L)	−0.141 (0.6927)	−0.278 (0.6999)	−0.130 (0.5928)
HDL (mmol/L)	0.079 (0.1508)	0.051 (0.1975)	0.042 (0.2250)
Free fatty acid (mmol/L)	−0.003 (0.2186)	−0.038 (0.2170)	−0.007 (0.2099)
Triglycerides (mmol/L)	0.037 (1.9900)	−0.379 (2.5157)	0.014 (0.8548)
Calcitonin (pmol/L)	−0.038 (0.479)	0.043 (0.113)	0.097 (0.237)

Data are mean (SD).
