# Supplementary material for: Efficacy and safety of once-weekly GLP-1 receptor agonist albiglutide (HARMONY 2): 52 week primary endpoint results from a randomised, placebo-controlled trial in patients with type 2 diabetes mellitus inadequately controlled with diet and exercise
Source: Diabetologia. 2015 Nov 17;59:266–74. doi: 10.1007/s00125-015-3795-1 (PMC4705137; doi:10.1007/s00125-015-3795-1)
Supplement: Supplementary file 7 — (PDF 28 kb) [file 125_2015_3795_MOESM7_ESM.pdf]

List of Participating Study Investigators by Country

Mexico: R Alvarado Ruiz, A Cabeza Gómez, JF Chávez Carbajal, JM Escalante Pulido, G González-Gálvez, MAS Madero Fernández del Castillo, NA Martinez Trejo, G Meléndez Mier, R Reyes Sánchez, J Rodriguez Saldaña, MC Saldate Alonso, LF Sánchez Arriaga, L Sauque Reyna, MA Tapia González

United States: L Akright, JLM Al-Amin, MA Allaw, DD Altamirano, LK Alwine, O Alzohaili, NS Andrawis, S Arora, S Babazadeh, JE Barrera, JL Beach, B Bergman, RH Bertenshaw Jr, K Blaze, D Bolton, S Brock, RE Broker, ME Brown, R Canadas, JP Capo Jr, CC Case, GH Cha, TA Christensen, LHS Chuck, CN Corder, FC Coulter, LI Cowan, MR Cox, H Cruz, N Daboul, RE De La Rosa, RG DeGarmo, JP Delgado, DS Denham, S Devenport, JD Diaz, MJ DiGiovanna, A Doshi, W Drummond, JB Eberly, JR Elsen, PE Emmans Jr, NR Farris, RD Ferrera, R Fillmore, BP First, N Fishman, D Fitz-Patrick, NW Gabra, R Gandolfi, MP Gilbert, RM Gilman, L Glaser, RM Glover II, D Grant, AJ Green, M Guice, PA Hartley, AJ Higgins, GT Hill Jr, EA Hubach, CR Hutchins, WP Jennings, AE Jimenez, SW Jones, V Kalen, JY Karl, AR Kelly, DM Kenton, CS Ketels, M Khan, CG Knopke III, SJ Kulback, JA LaFata, LA Landry, DC Larsen, G Ledesma, GA Ledger, JD Lentz III, GD Levinson, LS Levinson, MW Lin, RS Lipetz, ML Look, BC Lubin, KJ Lucas, B MacGillivray, HM Maldonado, L Maletz, JC Martinez, MJ McCartney, WC McKenzie, RA McLean, LT Megna, BH Merrick, DR Metz, BA Michlin, CI Mitchell, DJ Molter, CS Moorhead, MV Moro, JE Morriss III, PC Norwood Jr, MS Oberoi, S Ong, DK Pace, MA Pace, MV Pamganamamula, NA Patterson, PH Philander, RB Polakoff, LR Popeil, B Preston, RM Pucillo, KK Pudi, DS Ramstad, MS Rendell, JM Rhudy, KD Roberts, PD

Rosenblit, J Rosenstock, GE Ruoff, GB Ryckman, B Samuels, J Schmidt, MR Seidner, RJ Severance, SB Shachar, SA Smallow, TR Smith, JA Solomon, J Sparks, R Stegemoller, RB Stewart, D Stough, DA Streja, RJ Struble, RA Strzinek, D Sugimoto, RC Tanchanco, SO Teniola, TM Thorp, R Tidman, JM Tsao, P Waller, S Weakley, RL Weeks, S Weinrib, AJ Weisbrot, PN Weissman, JF Wilker, KL Wilks, DO Williams, AC Wine, E Wolfson, SM Zaidi, BM Zamora

Members of the Pancreatic Adjudication Committee

Firas Al-Kawas (Chairman; Georgetown University, Washington, DC, USA); Michelle Anderson (University of Michigan, MI, USA); and Robert Enns (University of British Columbia, BC, Canada).
